# Chemotherapy with ceramide in TNBC

**DOI:** 10.18632/oncoscience.196

**Published:** 2015-08-21

**Authors:** Patrick Legembre, Olivier Micheau, Bruno Ségui

**Affiliations:** Centre Eugène Marquis, rue bataille Flandres Dunkerque, Université de Rennes-1, INSERM ERL440, Equipe Labellisée Ligue Contre Le Cancer, rue bataille Flandres-Dunkerque, Rennes, France

**Keywords:** sphingolipids, EMT, CD95, TRAIL, metastasis

Metastasis remains the major cause of cancer-related mortality, and currently there is a lack of therapies to eliminate this risk. Therefore, it is of crucial interest to better understand this multi-step cellular process and find the best markers to identify patients who will develop early and distant relapse. This latter objective may allow clinicians to adapt their treatments and propose combination of therapeutic drugs to patients who are at the highest risk of metastasis.

CD95L belongs to the Tumor Necrosis Factor (TNF) superfamily and binds CD95 (also known as Fas). In contrast to its ubiquitously expressed receptor, CD95L exhibits a restricted expression pattern, being mainly found at the surface of activated T lymphocytes and natural killer (NK) cells where it contributes to the elimination of transformed and infected cells. Metalloproteases can cleave CD95L leading to its release in the bloodstream. While transmembrane CD95L (m-CD95L) is a potent apoptotic inducer that participates in immune surveillance and tolerance, metalloprotease-cleaved CD95L (cl-CD95L) fails to trigger apoptosis, but implements non-apoptotic signaling pathways (PI3K, K-Ras, NFkB, MAPK) promoting cell survival, proliferation and migration [1]. By doing so, CD95 behaves as an oncogene. We recently established that high concentrations of serum CD95L are present in triple negative breast cancers (TNBC). Among breast cancers, TNBCs are characterized by lack of estrogen and progesterone receptor expressions and absence of HER2 overexpression. Unlike others breast cancer categories, TNBC women do not benefit from targeted therapies and thereby, these patients relapse earlier than other breast cancer patients. Importantly, high amounts of cl-CD95L in TNBC women are associated with poor prognoses and administration of cl-CD95L in mice promotes the metastatic dissemination of TNBC cells [2].

Epithelial to mesenchymal transition (EMT) is a cellular reprogramming that plays pivotal role in embryonic development and that can be partly phenocopied in carcinogenesis, giving rise to aggressive tumors with increased metastatic capacity. EMT reprograms the apoptotic machinery in cancer cells to allow them to resist CD95-mediated cell death [3]. But not only, we recently demonstrated that EMT also enhances plasma membrane fluidity and thereby, promotes cell migration of breast cancer cells [4]. Sphingolipids (SLs) are sphingoid base-containing lipids, which are membrane components enriched in membrane microdomains, being concentrated into the outer leaflet of the plasma membrane and modulating its biochemical and biophysical properties such as membrane fluidity. EMT is associated with changes in SL metabolism, and some SL metabolites, such as gangliosides (*i.e.,* sialic acid-containing glycosphingolipids), can affect this cellular reprograming [5]. An integrative analysis of the gene expression in NCI-60 tumor cell lines revealed that the expression level of a ceramide synthase, namely ceramide synthase-6 (CerS6) decreases during EMT. CerS6 expression level mainly regulates the amount of C16:0-ceramide in cancer cells. Interestingly, whereas reducing the amounts of C16:0- ceramide in TNBC cells by down-regulating CerS6 expression level, increased plasma membrane fluidity and promoted cell motility, exogenous addition of non-cytotoxic doses of C16:0-ceramide or over-expression of CerS6 in TNBC cells led to a stiffening of plasma membrane, abrogating both basal and cl-CD95L-mediated cell migration [4]. These findings led us to postulate that administration of low doses (that may reduce side effects for the patients) of C16:0-ceramide in combination with chemotherapy in TNBC women may apply the brake on metastatic dissemination.

Like CD95L, tumor-necrosis-factor related apoptosis-inducing ligand (TRAIL) belongs to the TNF family. In line with CD95's pro-metastatic potential, DR5 (TRAIL-R2), one of the two agonistic TRAIL receptors, can promote breast cancer skeletal metastasis in a mouse xenograft model [6]. Earlier evidence already pointed to TRAIL promoting migration in TRAIL-resistant colon carcinomas [7]. More recently, it has been reported that DR5 displays pro-metastatic activity in KRAS derived non-small-cell lung cancers and pancreatic ductal adenocarcinoma (PDAC), and high expression levels of DR5 are correlated with increased invasion and reduced metastasis-free survival in KRAS mutated patients suffering from PDAC or colorectal cancer, respectively [8]. Although, regulation of SL membrane composition induced by TRAIL, is mostly ascribed to its pro-apoptotic potential, changes in SL content are also likely to contribute to regulation of DR5 pro-metastatic potential.

This growing body of evidence put forward that like TNF-R, CD95 and DR5 might correspond to pro-inflammatory receptors modulating the quantity and composition of SLs in cancer cells to affect the biophysical properties of their plasma membrane. These observations raise now the question of how the so-called “death receptors” can affect the SL metabolism to enhance cancer cell migration. Identification of the molecular mechanism linking these receptors to SL metabolism is likely to reveal new therapeutic options and targets to prevent tumor dissemination in cancers.
